# Genetic variations and alternative splicing: the Glioma associated oncogene 1, GLI1

**DOI:** 10.3389/fgene.2012.00119

**Published:** 2012-07-06

**Authors:** Peter G. Zaphiropoulos

**Affiliations:** Department of Biosciences and Nutrition, Karolinska Institutet,Huddinge, Sweden

**Keywords:** exon skipping, splice signal, splicing enhancer, splicing silencer, cryptic splice site

## Abstract

Alternative splicing is a post-transcriptional regulatory process that is attaining stronger recognition as a modulator of gene expression. Alternative splicing occurs when the primary RNA transcript is differentially processed into more than one mature RNAs. This is the result of a variable definition/inclusion of the exons, the sequences that are excised from the primary RNA to form the mature RNAs. Consequently, RNA expression can generate a collection of differentially spliced RNAs, which may distinctly influence subsequent biological events, such as protein synthesis or other biomolecular interactions. Still the mechanisms that control exon definition and exon inclusion are not fully clarified. This mini-review highlights advances in this field as well as the impact of single nucleotide polymorphisms in affecting splicing decisions. The Glioma-associated oncogene 1, GLI1, is taken as an example in addressing the role of nucleotide substitutions for splicing regulation.

## REGULATION OF SPLICING – *Cis* ELEMENTS, *TRANS* FACTORS

Alternative splicing has, in recent years, been demonstrated to occur in almost all intron-containing human genes (Pan et al., 2008; Wang et al., 2008). However, the regulatory mechanisms that control splice choices are not fully deciphered. Apart from the general problem of defining exonic sequences in a vast excess of intronic DNA (Schwartz et al., 2009a), a key issue concerns the mechanisms of the differential inclusion of exons in mature mRNAs. The prevailing views favor that this is controlled by the relative abundance of a limited number of splicing factors in a particular cell type (Chen and Manley, 2009). Additionally, alternative promoter usage may affect splicing decisions, as this can influence the recruitment of splicing factors or the elongation rate of RNA polymerase II (Kornblihtt, 2005; Muñoz et al., 2009). Furthermore, histone modifications are also thought to have a role in modulating splice choices (Luco et al., 2010). Moreover, the RNA secondary structure may affect splicing decisions (McManus and Graveley, 2011), with this claim being suggested more than 25 years ago (Solnick, 1985). In fact, the expansion of splicing factors, including the SR and hnRNP proteins, in higher eukaryotes (Busch and Hertel, 2012) is in agreement with the increased alternative splicing events in more complex organisms (Kim et al., 2007).

In addition to this *trans* factor dependent regulation of splice choices, *cis* sequences are also important in influencing splicing decisions, as these are thought to act as binding sites for the splicing factors. To start with, exons are flanked by the invariant intronic dinucleotides AG and GT, with the introns also characterized by a polypyrimidine track near their 3′ end and the branch point adenosine residue used for lariat formation during intron excision. However, it is obvious that additional *cis* sequence information is required in defining exons and influencing their inclusion in mRNAs. Such auxiliary sequences, which stimulate splicing are found in both exons (exonic splicing enhancers, ESE), and introns (intronic splicing enhancers, ISE; Aznarez et al., 2008; Lomelin et al., 2010; Brooks et al., 2011). Moreover, sequences that inhibit splicing have also been characterized, as exonic and intronic splicing silencers (ESS and ISS; Yu et al., 2008; Zhang et al., 2008; Wen et al., 2010). Additionally, the sequence complexity of these splicing regulatory elements is highlighted by the *in vivo* analysis of all possible hexamers as exonic splicing regulators, which revealed that out of the 4,096 combinations more than half could act as either ESE or ESS (Ke et al., 2011).

## SINGLE NUCLEOTIDE POLYMORPHISMS IN HUMAN GENOMES AND ALTERNATIVE SPLICING

The advances in sequencing technologies have made possible the complete sequence of individual human genomes at a rapid pace (Xia et al., 2012). Consequent is the expansion of the single nucleotide polymorphisms (SNP) entries in the dbSNP database, which currently reaches more than 40,000,000 validated RefSNPs^1^. Thus, on the average, a SNP is present in every 75 nucleotides within the three billion human genome sequence. Moreover, since the human protein-coding gene content is estimated to be 2% of the human genome, 800,000 SNPs can potentially influence gene expression and splicing, not to mention variations outside protein-coding genes that could alter gene regulation by mechanisms that are still poorly understood (Visel et al., 2009).

What characterizes the SNPs that modulate splicing? Firstly, exons that are known to be alternatively spliced, in contrast to constitutive exons, appear to have a higher proportion of SNPs that affect exonic splicing regulators. Additionally, ESSs are more frequently modified by SNPs than ESEs (de Souza et al., 2011). A systematic analysis of the SNPs that are known to affect splicing revealed a number of characteristics, including a predominance of ESS gains relative to ESE losses (Woolfe et al., 2010). These features have been introduced in a web-based tool allowing the prediction of the impact of SNPs on alternative splicing regulation, the Skippy software^2^. In this context it is worth noting that SNPs that affect the strength the 5′ splice site, but not the conserved GT dinucleotide, are compensated by other regulatory signals, resulting in minimal changes in splicing, arguing in favor of a certain robustness in maintaining splicing patterns (Lu et al., 2011).

## MUTATIONS AND GENE FUNCTION

It has generally been assumed that exonic mutations (the term mutation refers to a polymorphism occurring in less than 1% of the population) affect the protein product of the corresponding gene by introducing a stop codon (non-sense mutation), by altering an amino acid (missense mutation) or by changing the translation reading frame (frameshift mutation). However, this postulate had been challenged by the finding that nucleotide substitutions that do not change the encoded amino acid (synonymous mutation) may also alter protein structure, through an indirect way, namely by modulating splicing patterns (Chamary et al., 2006). This can be rationalized by either an activation of a cryptic splice site, as in the case of the LMNA gene (Eriksson et al., 2003), or the presence of ESE and ESS motifs, which are affected by the introduction of a synonymous nucleotide substitution and consequently may alter the canonical splicing pattern. In fact, it has been convincingly argued that the genetic code can effectively carry additional information, such as splicing signals, besides its capacity to encode amino acids (Itzkovitz and Alon, 2007). Moreover, an analysis of 27,681 non-sense and missense disease-causing mutations revealed that about 25% of these actually change exonic splicing regulators, providing evidence that alterations of splicing patterns is not a sole characteristic of synonymous substitutions (Sterne-Weiler et al., 2011).

## THE GLIOMA-ASSOCIATED ONCOGENE 1, GLI1 – ALTERNATIVE SPLICING

**FIGURE 1:**
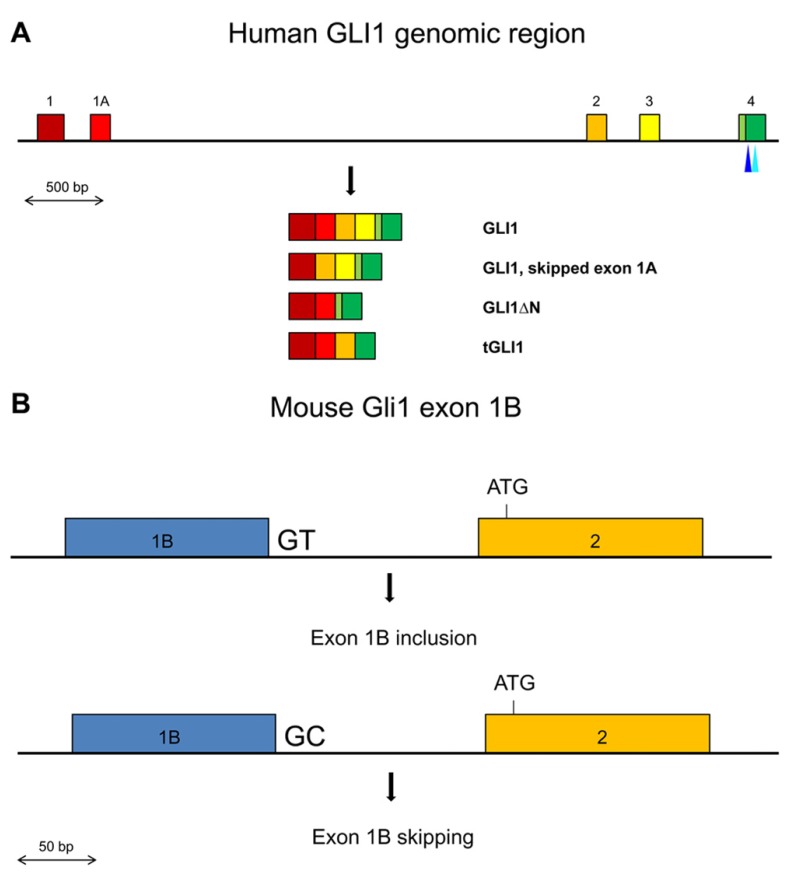
**(A)** Schematic representation of the exons 1 to 4 region in the human GLI1 gene. Exons are shown by colored boxes. The GLI1 mRNA variants are indicated. The canonical translation initiation codon is present in exon 2. In GLI1ΔN, skipping of exons 2 and 3 results in the use of a methionine codon in exon 4 as the initiator. In tGLI1, skipping of exon 3 and partially exon 4 does not alter the translational reading frame. The positions of the SNPs in exon 4 that may affect exon skipping or generate an ectopic splice site are indicated by light and dark blue triangles respectively. **(B)** Schematic representation of the Gli1 exon 1B inclusion/skipping in the mouse. BALB/c mice contain the conserved GT dinucleotide at the exon 1B/intron 1B junction resulting in exon 1B being mostly included in the Gli1 mRNAs. C57BL/6 mice contain a GC dinucleotide at the exon 1B/intron 1B junction resulting in exon 1B being mostly skipped in the Gli1 mRNAs.

The GLI1 oncogene encompasses about 12 kb of genomic sequences in chromosome 12q13.2–q13.3 and is composed of two 5′ non-coding exons, exons 1 and 1A, and 11 coding exons, exons 2 to 12. Two splice variants have been identified in human tissues that affect coding exons, GLI1ΔN, which lacks exons 2 and 3 (Shimokawa et al., 2008), and tGLI1, which lacks exon 3 and part of exon 4 (Lo et al., 2009). However, variants that do or do not include the non-coding exon 1A have also been described (Wang and Rothnagel, 2001; **Figure 1A**).

In the mouse, the Gli1 gene contains a third non-coding exon, exon 1B (**Figure 1B**). Interestingly, the 5′ splice site of exon 1B in the BALB/c mouse strain contains the canonical GT dinucleotide, while in the C57BL/6 mouse strain this is substituted with GC. The splice signal alteration confers a preferential inclusion of exon 1B in BALB/c mice resulting in a longer 5′ UTR region. Moreover, this increased 5′ UTR length was found to have a negative impact on the translation efficiency of the Gli1 mRNA, with a consequent reduced amount of synthesized protein (Palaniswamy et al., 2010). These observations argue that SNPs that affect splicing may not necessarily alter the primary structure of the protein product but can also have an impact on the amount of produced protein, as a result of changes in the length/structure of the 5′ UTR region.

## THE GLIOMA-ASSOCIATED ONCOGENE 1, GLI1 – SNPs

The dbSNP database revealed that there are 249 variants in the human GLI1 gene sequence (May 2012). From these 127 are intronic, 32 are synonymous, 59 are non-synonymous, and 14 are in the UTR regions.

By centering on the genomic region that engages in alternative splicing of protein-coding exons, namely exons 2 to 4 and the 100 bases flanking sequence, 27 SNPs, with four being synonymous and four non-synonymous, were identified. Moreover, the use of the Skippy software revealed that the highest score for exon skipping of these eight exonic SNPs is for the C to T missense rs143548857 in exon 4. Direct experimentation will be required to examine whether this predicted impact on exon 4 splicing is indeed taking place, as this will introduce a translational frameshift.

From the 36 synonymous and non-synonymous SNPs in the remaining protein-coding internals exons, namely exons 5 to 11, only the C to T synonymous rs7973381 in exon 8 has an equally high score for exon exclusion as rs143548857. Interestingly, the size of exon 8 is 150 nucleotides, highlighting that potential exon skipping will not initiate non-sense mediated decay (Durand and Lykke-Andersen, 2011).

Since in the natural tGLI1 variant an alternative 3′ splice site is used in exon 4 (**Figure 1A**), SNPs that could potentially mediate activation of ectopic splice sites in exon 4 were examined using the Skippy software. Interestingly, the G to A synonymous rs139988338 in exon 4 creates a 3′ ectopic splice site with a high score. Moreover, none of the SNPs in the remaining internal exons of GLI1 create a 3′ ectopic splice site that has a higher score. Worth noting is that the strength of the rs139988338-mediated 3′ ectopic splice site in exon 4 is, in fact, higher than the natural tGLI1 alternative 3′ splice site, according to the MaxEnt splice site scoring program^3^.

Additionally, in the non-coding but alternatively spliced exon 1A three SNPs are identified, rs10783826, rs10783827, and rs118093490. These SNPs cannot effectively be evaluated by the Skippy software, as the program is not optimized for non-coding exons (Laura Elnitski, personal communication). Moreover, exon 1A is not fully annotated in the human Genome Build 37, which automatically excludes it from the software analysis, since only annotated exons can be used. On the other hand, manual inspection of the elicited base substitution by the three SNPs and taking advantage of the published strength of all hexamers as ESEs and ESSs (Ke et al., 2011) revealed that for rs10783826 and rs118093490 the nucleotide change increased the impact of the corresponding hexamer as a silencer, while for rs10783827 the change was in favor of an enhancer. These findings were observed irrespective of whether the hexamers were shifted by one base so the SNP was placed at either the third or the fourth nucleotide position within the hexamer. Moreover, the SROOGLE software^4^ (Schwartz et al., 2009b), which uses additional programs to identify ESEs and ESSs, revealed a similar trend. Thus, it is possible that the differential inclusion of exon 1A may be dependent of these SNPs, and the resulting variable length of the 5′ UTR region could have an impact on the amount of the translated protein product, as previously seen with the mouse Gli1.

## CONCLUSION

Alternative splicing is a major post-transcriptional mechanism that diversifies gene expression. In addition to *trans* acting factors, *cis* regulatory sequences have also an impact in controlling splice choices, highlighting the potential significance of SNPs in modulating splicing decisions. SNPs that affect splicing may be present in both intronic and exonic regions, mapping at regulatory elements such as splicing enhancers and silencers. Moreover, for exonic SNPs, not only synonymous but also missense and non-sense SNPs can influence splice choices, providing evidence that the major biological impact of some of these exon variants may be on mRNA splicing rather than on protein synthesis.

Using the Glioma-associated oncogene 1, GLI1, as an example, SNPs are predicted to have a role in controlling splicing patterns. Interestingly, GLI1 exons that are known to engage in alternative splicing appear to preferentially encompass SNPs that may affect splice choices. Finally, although user-friendly prediction programs simplify the analysis of the role of SNPs in splicing regulation, the requirement of direct experimentation has not been fully substituted.

## Conflict of Interest Statement

The author declares that the research was conducted in the absence of any commercial or financial relationships that could be construed as a potential conflict of interest.

## Footnotes

1 http://www.ncbi.nlm.nih.gov/projects/SNP/snp_summary.cgi

2 http://research.nhgri.nih.gov/skippy

3 http://genes.mit.edu/burgelab/maxent/Xmaxentscan_scoreseq_acc.html

4 http://sroogle.tau.ac.il
